# Prognostic Importance of Cell Cycle Regulators Cyclin D1 (*CCND1*) and Cyclin-Dependent Kinase Inhibitor 1B (*CDKN1B*/p27) in Sporadic Gastric Cancers

**DOI:** 10.1155/2016/9408190

**Published:** 2016-10-03

**Authors:** Petra Minarikova, Lucie Benesova, Tereza Halkova, Barbora Belsanova, Inna Tuckova, Frantisek Belina, Ladislav Dusek, Miroslav Zavoral, Marek Minarik

**Affiliations:** ^1^Department of Internal Medicine, 1st Faculty of Medicine, Charles University and Military University Hospital, 169 02 Prague, Czech Republic; ^2^Center for Applied Genomics of Solid Tumors, Genomac Research Institute, 161 00 Prague, Czech Republic; ^3^Department of Pathology, Military University Hospital, 169 02 Prague, Czech Republic; ^4^Department of Surgery, 2nd Faculty of Medicine, Charles University and Military University Hospital, 169 02 Prague, Czech Republic; ^5^Institute for Biostatistics and Analyses, Faculty of Medicine, Masaryk University, Brno, Czech Republic

## Abstract

*Background*. Gastric cancer is known for a notable variety in the course of the disease. Clinical factors, such as tumor stage, grade, and localization, are key in patient survival. It is expected that molecular factors such as somatic mutations and gene amplifications are also underlying tumor biological behavior and may serve as factors for prognosis estimation.* Aim*. The purpose of this study was to examine gene amplifications from a panel of genes to uncover potential prognostic marker candidates.* Methods*. A panel of gene amplifications including 71 genes was tested by multiplex ligation-dependent probe amplification (MLPA) technique in 76 gastric cancer samples from a Caucasian population. The correlation of gene amplification status with patient survival was determined by the Kaplan-Meier method.* Results*. The amplification of two cell cycle regulators,* CCND1* and* CDKN1B*, was identified to have a negative prognostic role. The medial survival of patients with gastric cancer displaying amplification compared to patients without amplification was 192 versus 725 days for* CCND1 *(*P* = 0.0012) and 165 versus 611 days for* CDKN1B* (*P* = 0.0098).* Conclusion*. Gene amplifications of* CCND1* and* CDKN1B* are potential candidates to serve as prognostic markers for the stratification of patients based on the estimate of survival in the management of gastric cancer patients.

## 1. Introduction

Gastric cancer is a multifactorial disease resulting from a multistep process in which both exogenous and endogenous factors take place. Exposition to nitrates/nitrites, excess salt in the diet, lack of fresh vegetables and fruits, and the associated deficiency of vitamin C along with* Helicobacter pylori* infection leading to premalignant conditions such as chronic atrophic gastritis and intestinal metaplasia are the most prominent exogenous factors [1–3]. The diagnosis of gastric cancer is difficult, mainly due to a lack of early symptoms, which are often very nonspecific. The disease is therefore, in most cases, diagnosed at an advanced stage when treatment is limited and the 5-year survival rate is only 10–30% [4]. Furthermore, the heterogeneity of gastric tumors has resulted in significant differences in patient survival.

An established histopathological evaluation of gastric carcinomas recognizes distinct subtypes according to an original 1965 publication by Lauren [5]. The two types, a well-differentiated intestinal type and an undifferentiated diffuse type, appear to arise from different developmental pathways [6]. Accordingly, there is also overwhelming evidence of the diverse molecular mechanisms as likely being the main factor causing the different clinical behavior of the two Lauren subtypes [7]. While the intestinal type follows the classic “colorectal” pattern of metastatic invasion into the liver and/or the lungs, the diffuse type exhibits more aggressive growth with a risk of bone metastases and/or peritoneal spread [8].

An exact course of the development and progression of gastric cancer at the molecular level is yet to be fully described in detail, but research in the fields of molecular epidemiology and cancer genomics has helped to clarify some of the basic characteristics [9]. The current model describes genetic defects accompanying the transformation of gastric tissue in different tumor types under the intermediate stage of chronic atrophic gastritis and intestinal metaplasia [10].* Helicobacter pylori* (HP), which colonizes gastric mucosa and contributes to the development of chronic gastritis infection, is a known factor involved in the initiation of the gastric cancer process [3]. Although the prevalence of HP infection in patients with gastroduodenal ulcer disease is approximately 70% (depending on age), it is still very common also in the general population (24–84%) [11–13]. Another important factor in gastric cancer is Epstein-Barr virus (EBV) infection. One of the proliferation mechanisms of this oncogenic herpesvirus is the increased production of antiapoptotic Bcl-2 protein in host cells allowing their subsequent malignant transformation [14]. Given the high prevalence of both pathogens in a healthy population, the role of other exogenous and endogenous factors in developing this disease is evident.

According to the National Cancer Institute, gastric cancer historically ranks among the worst malignancies in prognosis, with less than 30% of patients surviving for 5 years after diagnosis [15]. A significant improvement in patient survival resulted from the arrival of the first gastric cancer biological therapy in 2010, when trastuzumab was approved by the FDA (Food and Drug Administration) for a subset of metastatic cancers overexpressing HER2 [16]. Gastric tumors exhibiting the overexpression/amplification of HER2 have a priori worse prognosis, but when treated by trastuzumab patients can achieve clinically significant stabilization or remission of the disease. As a result, HER2 overexpression and amplification had since become a key predictive and prognostic marker in targeted gastric cancer therapy [17].

Testing for the presence of* HER2 (ERBB2)* gene amplification for the prediction of the tumor response to anticancer treatment by anti-ERBB2 monoclonal antibody has fundamentally transformed the oncology of gastric cancer [18]. However, the presence of* HER2* amplification is in only about 20% of gastric cancers, prompting search for other suitable molecular predictors and prognostic markers useable for the disease management. With the recent development in the area of multipanel sequencing using next-generation technologies, a number of reports have described a comprehensive mapping of somatic mutations in various solid cancers [19]. The studies have identified the vast majority of single nucleotide variants (point mutations) and short insertions/deletions. In gastric cancer, this has recently led to the identification of molecular subtypes showing distinct patterns of origin and resulting in different clinical manifestation and prognosis [20]. In addition to these frequent aberrations, a considerable contribution from large copy number variations, including gene amplifications and/or deletions, has long been documented [19, 21] and extensively studied [22–26]. A recent study has identified molecularly distinct subtypes by evaluating aberrant deletion of amplification in key receptor tyrosine kinases (RTKs). Subsequently, several RTKs have been identified as drivers of tumor development in gastric cancers including HER2/ERBB2, VEGFR, PDGFR, FGFR, IGFR, and Met [27].

As a result of the prominent incidence of gastric cancer in Asian [28] and Latin American [29] countries, most studies directed at molecular characterization have been conducted on Asian populations. In this work, we present results from a European (Caucasian) population of gastric cancer patients. We employ the multiplex ligation-dependent probe amplification (MLPA) technique [25] to scan for amplifications in a set of 71 genes. We then present the correlation of molecular data with clinical characteristics such as the tumor stage and location and, most importantly, the patient's survival.

## 2. Materials and Methods

The study was approved by the Scientific and Ethics Boards of the Military University Hospital. A total of 76 patients (all European Caucasians) with clinically confirmed gastric cancer were included in this study, along with relevant clinical data including disease stage, Lauren type, tumor localization, and overall survival (see Table 1). The samples were obtained either as endoscopic biopsies or as peroperative resections in a form of formalin-fixed paraffin-embedded (FFPE) sections (*n* = 60) or in a form of fresh frozen tissue (*n* = 16). Genomic DNA was extracted from FFPE sections and fresh samples using a commercial kit (JetQuick Tissue DNA Spin, GENOMED GmbH, Löhne, DE) based on a spin-column protocol. A panel of gene amplifications including 71 genes taking part in the major processes of cancer proliferation (division and growth), apoptosis, and metastasis was tested by multiplex ligation-dependent probe amplification (MLPA). A combination of commercial MLPA kits was used (SALSA MLPA P144, SALSA MLPA P145, and SALSA MLPA P175, MRC Holland, NL), and the procedure was performed according to the manufacturer's instructions. Briefly, 5 *μ*L of extracted DNA was incubated for 5 minutes at 98°C, and then 3 *μ*L of MLPA hybridization master was added and the mixture was first briefly (1 minute) incubated at 98°C and then left for hybridization at 60°C for 16–20 hours. The next step was the addition of 32 *μ*L of MLPA ligase master and incubation at 54°C for 15 minutes and at 98°C for 5 minutes. Finally, 10 *μ*L of polymerase master was added to the mixture and the PCR reaction was performed using 35 cycles each with 30-second denaturation at 95°C, 30-second annealing at 60°C, and 60-second extension at 72°C. The final PCR product mixture was then purified by sephadex spin columns (AutoSeq G-50, GE Healthcare Biosciences, Piscataway, NJ), mixed in a 3 : 1 ratio with a fluorescent size standard (GeneScan 500 ROX, ThermoFisher Scientific, Grand Island, NY), diluted 10-fold in Hi-Di Formamide (Sigma-Aldrich, St. Louis, MO), and analyzed on a 16-capillary DNA analyzer (ABI PRISM 3100) in POP-7 matrix on a 36 cm capillary array with 15 seconds at 1.6 kV injection and 15 kV run voltage at 60°C separation temperature.

The MLPA electropherograms were evaluated by GeneMarker software using MLPA panels (available for download from SoftGenetics website). The panel containing multiple receptor tyrosine-kinases families as well as other genes involved in cell cycle and proliferation was subjected to evaluation of gene amplifications. In accordance with previous reports [23, 25], the result was determined by the peak ratio of the evaluated gene probe to a control probe (or a reference probe), where a value below 0.7 defined a marker as lost, a value between 0.7 and 1.5 defined a marker as normal, and a value of more than 1.5 marked the evaluated gene as amplified. In some instances, multiple probes were present for a given gene and the amplification was only assigned if more than 50% of the probes were above the 1.5 threshold.

Patient survival analysis was done using the Kaplan-Meier method using MedCalc statistical software (MedCalc, Oostende, Belgium).

## 3. Results and Discussion

The overall success rate of DNA extraction from the gastric FFPE samples was at a level of 85%. This was mainly due to gastric biopsies processed into FFPE sections whose yields were at 70%, primarily due to low quality and, eventually, low amounts of DNA.

### 3.1. Evaluation of Gene Amplification by MLPA Technique

**Figure 1:**
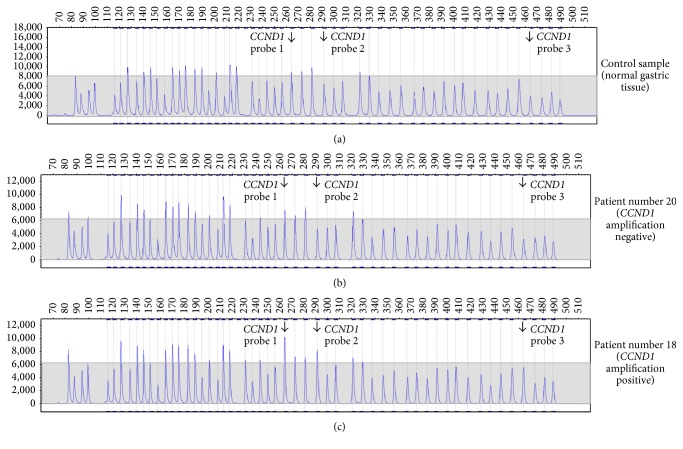
Evaluation of* CCND1* gene amplification by MLPA. Normal tissue (control) sample (a), carcinoma samples without amplification (b), and carcinoma samples with amplification (c). Evaluation based on 3 different* CCND1* probes.

**Figure 2:**
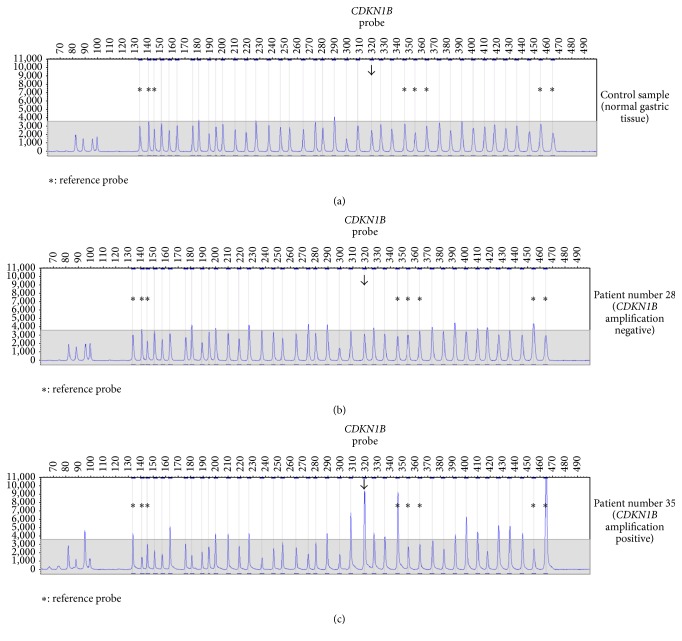
Evaluation of* CDKN1B *gene amplification by MLPA. Normal tissue (control) sample (a), carcinoma samples without amplification (b), and carcinoma samples with amplification (c). Evaluation against a set of external control probes (denoted by asterisk).

MLPA technique has previously been applied for evaluation of* HER2* gene amplification in breast cancer [30, 31] as well as* EGFR* gene amplification in lung cancer [32, 33]. More recently, the approach has been validated for testing also in gastric cancer [25, 34]. In this work, gene amplification has been evaluated with the use of dedicated software (GeneMarker) and according to previously validated procedures (see Section 2). Since the MLPA evaluation is based on quantitative reading of peak intensities, it is, naturally, influenced by the level of background set by the presence of nonmalignant components in the tested sample. Therefore, tumor cellularity is essential as reported recently [35]. The representative results of MLPA evaluation of gene amplification in gastric carcinoma tissue are shown in Figures 1 and 2.  Figure 1 depicts MLPA results observed for normal gastric tissue acting as control (a), gastric cancer tissue without* CCND1* amplification (b), and gastric cancer tissue exhibiting (among other genes) amplification of* CCND1* detected clearly for 2 out of 3* CCND1* probes (c). In a similar manner, Figure 2 illustrates MLPA result for evaluation of* CDKN1B *amplification with the normal nonmalignant tissue (a), amplification negative tumor (b), and amplification positive tumor (c), respectively. In case of* CDKN1B*, only a single MLPA probe was available.

Because there is a possible variability in degradation of DNA within a single FFPE sample, the consistency of the results was finally tested. A separate round of repetitive experiments was performed for a small subgroup of 13 randomly chosen FFPE samples starting again from the original tissue material resulting in 100% concordance for both* CCND1* and* CDKN1B*.

### 3.2. Amplification of* CCND1* Gene

Multifactorial analysis did not reveal any correlation between any of the tested genes and patient gender, tumor localization (cardia, body, and antrum), or the Lauren histological subtype (intestinal, diffuse) that would bear a statistical correlation. The amplification rate of* HER2* was 20.4% (11/54), which is in agreement with frequently reported FISH results [36, 37]. Univariate analysis of survival has returned two genes,* CCND1* and* CDKN1B*, whose amplification status was related to overall patients survival.

Cyclin D1, encoded by* CCND1* gene, is a member of a family of enzymes that stimulate the progression of the cell through the cell cycle. Cyclins serve as activators when bound to a complex with cyclin-dependent kinases (Cdk). Cyclin D1 regulates the progression from G1 to the S phase of the cell cycle. Its production is induced in the G1 phase by MAPK/ERK (Ras/Raf) signaling [38] stimulated upon binding of growth factors to a number of receptor tyrosine kinases (RTKs). In some reports, the amplification of* CCND1* in gastric cancer has been put in direct correlation to the amplification of some of the RTKs. Overexpression or amplification of cyclin D1 is acknowledged as an early causative event in the tumorigenesis of many solid tumors [39–41]. More recently,* CCND1* expression has been negatively linked with the survival of resected gastric cancer patients [42]. Finally, the importance of RTKs/CCND1 in gastric cancer has also been recognized as an indication of chromosomal instability (CIN) phenotype, which is detectable in several of the recently introduced molecular subgroups [20].

**Figure 3:**
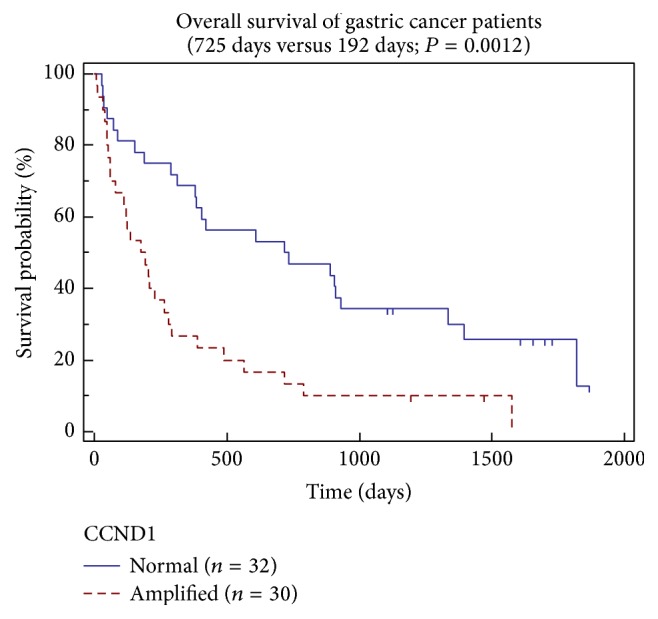
Kaplan-Meier curves for overall survival of gastric cancer patients with tumors with and without* CCND1* gene amplification. A statistical difference in the median survival has been reached at 192 days versus 725 days (*P* = 0.0012).

Amplification of* CCND1* gene has previously been found in gastric cancers [19, 22, 25, 26, 43]. In our patient cohort, we detected* CCND1* gene amplification in 49.2% of the tumors (31/63), evenly distributed throughout the gastric cardia, body, and antrum. The Kaplan-Meier plot for the overall survival of patients with or without the* CCND1* gene amplification is shown in Figure 3. A group of patients with gastric carcinomas displaying* CCND1* gene amplification (*n* = 30) had median overall survival of just 192 days, while the overall median survival in a group of patients whose tumors did not show* CCND1 *amplification (*n* = 32) was 725 days. The results are statistically significant with *P* = 0.0012. The validity of the independence of the* CCND1* prognostic value is further supported by the structure of both groups, as in both groups there were patients in all stages of the disease as well as patients undergoing surgical treatment.

### 3.3. Amplification of* CDKN1B* Gene

The Cyclin-Dependent Kinase Inhibitor 1B gene (*CDKN1B*) encodes a cyclin-dependent kinase inhibitor protein referred to as p27 (or p27^Kip1^). One of its functions is to block the activation either of cyclin D itself or of a complex of cyclin D with cyclin-dependent kinase (Cdk4) complexes, hence ceasing the cell cycle progression [44]. The native function of* CDKN1B* is therefore tumor suppression, and there are reports of the reduced expression levels of p27 as a negative prognostic marker in various types of malignancies, including gastric cancer [45–47]. There is, however, a completely different mechanism involving p27 protein as a promotor of metastasis. The somewhat unusual functional dichotomy arises from the mislocalization of nuclear p27 into the cytoplasm by phosphorylation or activation by MAPK and PIK3CA oncogenic pathways [48–50]. The mislocalized cytoplasmic p27 acquires an oncogenic role to promote cell migration, invasion, and metastasis [51]. Furthermore, it was recently reported that* Helicobacter pylori* may be responsible for the mislocalization of p27 in gastric cancer [52].

**Figure 4:**
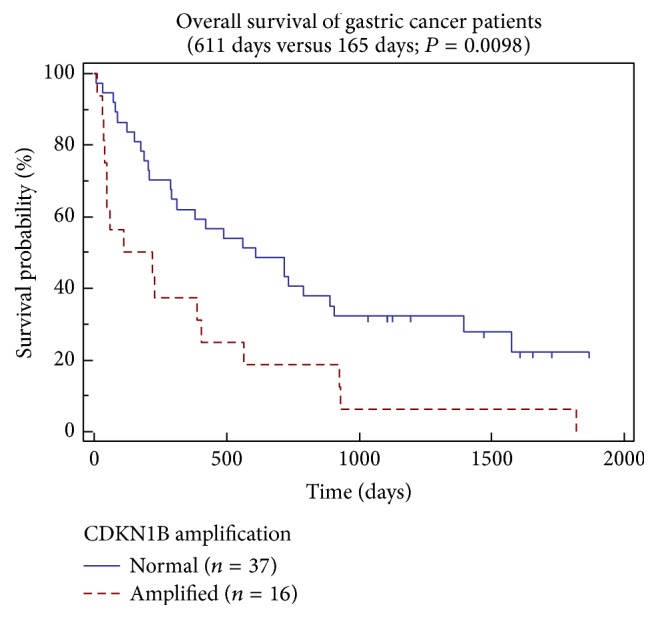
Kaplan-Meier curves for overall survival of gastric cancer patients with tumors with and without* CDKN1B *(P27^Kip1^) gene amplification. A statistical difference in the median survival has been reached at 165 days versus 611 days (*P* = 0.0098).

In our study, amplification of* CDKN1B* gene was detected in 31.5% (17/54) of gastric carcinomas. Also here, carcinomas exhibiting the* CDKN1B* gene amplification were detected in all parts of the stomach. The Kaplan-Meier analysis revealed a statistically significant difference (*P* = 0.0098) in the overall survival of patients with or without the* CDKN1B* gene amplification (see Figure 4). A group of patients with gastric carcinomas displaying* CDKN1B *gene amplification (*n* = 16) had median overall survival of 165 days, while a group of patients whose tumors did not show* CDKN1B *amplification (*n* = 37) had median overall survival of 611 days.

The presence of specific gene amplification, resulting from aberrant activation of various signaling pathways, indicates distinct tumor characteristics that may lead to specific tumor behavior. Genes identified in this study,* CCND1* and* CDKN1B*, are closely involved in a number of cellular processes that are central to the initiation and proliferation of gastric cancer. In addition to common carcinogenic pathways, including chromosomal or microsatellite instability, both genes take part in a system of complex interactions between RTK-induced signaling pathways and cell cycle regulation [53]. Aberrations of this system have been recognized as key signatures in the classification of gastric cancer subtypes [21]. In addition, general coexistence of aberrant* CCND1*/*CDKN1B* genes with a variety of mutated tumor suppressors and oncogenes is frequently found in solid cancers as recently reported [54]. In addition to their prognostic role demonstrated in this article, gene amplifications of* CCND1* and* CDKN1B* may also be useful in the prediction of gastric cancer therapy response, as both genes have been recognized as important biological targets [40, 55].

## 4. Conclusion

By evaluating the amplification status of 71 genes in mainly advanced gastric cancers from a Caucasian population of patients and by its correlation with patient survival, two members of the cell cycle regulation system,* CCND1* and* CDKN1B*, were identified as potential candidates for assessment of prognosis. The overall amplification rates found in the present work were higher when compared to previous reports [22, 25, 26]. This can be attributed, in part, to differences in the base characteristics of the tested populations, most importantly the race and living environment resulting in disparity in gastric cancer epidemiology [56]. Furthermore, as the disease stage seems to play a fundamental role in the occurrence of gene amplifications, cohorts of patients undergoing surgical therapies with a significant portion of early stage cancers from other studies may exhibit lower rates [57]. The result of this project may lead to further studies directed at cell cycle control in finding predictors and prognostic markers in the management of gastric cancers.

## Figures and Tables

**Table 1:** Patients' characteristics.

*Gender*	
Women	31
Aged	34–98 (median 67.7)
Men	45
Aged	40–89 (median 68.0)
*Localization*	
Cardia	15
Body	34
Antrum	27
*Stage*	
I	7
II	9
III	20
IV	40
*Lauren type*	
Intestinal	47
Diffuse	29
*Treatment*	
Undergoing surgery	29
Inoperable	47
*CCND1 status*	
Amplified	31
Normal	32
*CDKN1B status*	
Amplified	17
Normal	37
